# Improved Draft Genome Sequence of Pseudomonas poae A2-S9, a Strain with Plant Growth-Promoting Activity

**DOI:** 10.1128/MRA.00275-19

**Published:** 2019-04-11

**Authors:** Ye Xia, Seth DeBolt, Qin Ma, Adam McDermaid, Cankun Wang, Nicole Shapiro, Tanja Woyke, Nikos C. Kyrpides

**Affiliations:** a Department of Plant Pathology, The Ohio State University, Columbus, Ohio, USA; b Department of Horticulture, University of Kentucky, Lexington, Kentucky, USA; c Department of Biomedical Informatics, The Ohio State University, Columbus, Ohio, USA; d Department of Agronomy, Horticulture, and Plant Science, South Dakota State University, Brookings, South Dakota, USA; e Department of Energy Joint Genome Institute, Walnut Creek, California, USA; Indiana University, Bloomington

## Abstract

We report here the improved draft genome sequence of Pseudomonas poae strain A2-S9, a bacterium that was originally isolated from switchgrass plants and exhibited the capacity for plant growth promotion. Its genome has a size of 6.68 Mbp and a GC content of 61.3%. The genome encodes 6,022 predicted protein-coding genes.

## Abstract (precis)

We report here the improved draft genome sequence of Pseudomonas poae strain A2-S9, a bacterium that was originally isolated from switchgrass plants and exhibited the capacity for plant growth promotion. Its genome has a size of 6.68 Mbp and a GC content of 61.3%.

## ANNOUNCEMENT

The plant-associated microbiome performs important functions for plant growth, development, and health (1, 2). These microbes could be beneficial for plant biomass production and sustainable agriculture (3). Pseudomonas is a genus of Gram-negative, rod-shaped bacteria. They are among the most representative beneficial microbes associated with diverse plant species and have been documented to enhance plant growth and resistance to biotic and abiotic stresses (4, 5). Pseudomonas poae strain A2-S9 was originally isolated from switchgrass plants (Panicum virgatum), one of the most important biofuel crops, grown on a reclaimed coal-mining site in western Kentucky (6, 7). Through prior greenhouse experiments, it was found that Pseudomonas poae strain A2-S9 (originally named Pseudomonas sp. strain 47A) could promote switchgrass plant (Panicum virgatum cv. Alamo) growth (6). Therefore, it may have potential to improve the fitness of biofuel crops under stressful environmental conditions. The aim of this study was to obtain the genome of Pseudomonas poae strain A2-S9 to provide some insight into the metabolic and molecular mechanisms of the beneficial interactions between this strain and its switchgrass plant host.

The switchgrass plants were collected from a coal-mining site in Kentucky (6). The samples from switchgrass shoots and roots were cut into 1- to 1.5-cm segments, surface sterilized with 20 to 30% bleach, and further washed (3 to 5 times) with sterilized water. Then, the samples were put on plates with tryptic soy agar (TSA) medium (Sigma, USA) and incubated in a 26°C incubator for 3 to 5 days. Different bacterial isolates emerging from the plant segments were further isolated and purified (3 times) on TSA plates. Among these isolates, a single colony of Pseudomonas poae strain A2-S9 was cultured in tryptic soy broth (TSB) medium (Sigma, USA) at room temperature for 1 to 2 days (6). The bacterial cells were then centrifuged and pelleted for DNA extraction. The genomic DNA was extracted using the cetyltrimethylammonium bromide (CTAB) approach developed by the U.S. Department of Energy Joint Genome Institute (DOE-JGI; https://jgi.doe.gov/user-program-info/pmo-overview/protocols-sample-preparation-information/jgi-bacterial-dna-isolation-ctab-protocol-2012). The genomic DNA was sequenced by Pacific Biosciences (PacBio) technology with 86× depth at DOE-JGI (8), which generated 282,673 filtered subreads totaling 771.7 Mbp. The raw reads were assembled using Hierarchical Genome Assembly Process (HGAP) v. 2.3.0 (9). The average length of reads of >5 kb was 8,467 bp for raw reads and 7,681 bp for filtered subreads, respectively. Genome annotation was carried out by using the JGI Integrated Microbial Genome (IMG) system (10). The genes were identified using Prodigal v. 2.5 (11). The default parameters were used for all software except where otherwise specified.

**FIG 1:**
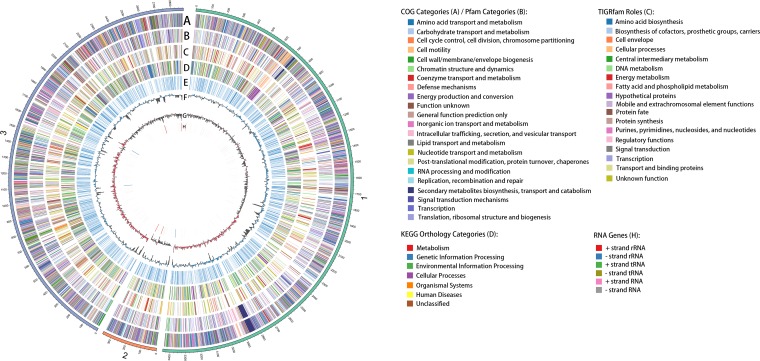
Circular view of the three contigs of the Pseudomonas poae A2-S9 genome. The circles from outside to inside denote (i) protein-coding genes with clusters of orthologous groups (COGs), (ii) protein-coding genes with Pfam, (iii) protein-coding genes with TIGRFAMs, (iv) Kyoto Encyclopedia of Genes and Genomes (KEGG) orthology regions, (v) transmembrane helix regions, (vi) GC content (blue indicates above and black indicates below the genome average of 61.3%, 5-kb window), (vii) GC skew (red indicates above and black indicates below zero, 5-kb window), and (viii) RNA genes. The figure was generated using the software Circos (12).

The total genome size of Pseudomonas poae strain A2-S9 is 6.68 Mbp with 3 contigs (Fig. 1). The GC content of this strain is 61.3%. There are 6,022 protein-coding genes, 5,114 of which have function predictions. The genome of strain A2-S9 encodes 415 genes involved in biosynthetic clusters and 731 genes involved in coding signal peptides. A total of 190 RNA genes were identified, including 22 rRNA genes, 70 tRNA genes, and 98 other RNA genes. Of the 22 rRNA genes, 7 are 5S rRNA, 7 are 16S rRNA, and 8 are 23S RNA (Fig. 1).

The genome information of Pseudomonas poae strain A2-S9 will be a critical resource for studying further the functional potential of this organism and for the application of this beneficial bacterium in enhancing switchgrass yield for biofuel production.

### Data availability.

The whole-genome sequence described here has been deposited at DDBJ/EMBL/GenBank under BioProject accession no. PRJNA243959, NCBI BioSample accession no. SAMN02745526, NCBI SRA accession no. SRS1644721, and GOLD Project identifier Gp0060935. The sequence described in this paper is the first version. The associated sequence data can also be found at the Joint Genome Institute (JGI) portal under the IMG taxon oid no. 2603880217 (https://genome.jgi.doe.gov/portal/PsefluA2S9_FD/PsefluA2S9_FD.info.html).
